# Portal hypertension: An uncommon presentation of Caroli's syndrome

**DOI:** 10.1002/ccr3.3374

**Published:** 2020-09-24

**Authors:** Akash Raut, Suraj Shrestha, Sushan Homagain, Amar Jayswal, Bikal Ghimire

**Affiliations:** ^1^ Maharajgunj Medical Campus Institute of Medicine Kathmandu Nepal; ^2^ Department of Gastrointestinal Surgery Tribhuvan University Teaching Hospital Kathmandu Nepal

**Keywords:** Caroli's syndrome, hematemesis, pediatrics, portal hypertension

## Abstract

Portal hypertension is not a classical presentation of Caroli's syndrome. However, some young children can present with overt signs and symptoms indicative of advanced disease state despite the improvement in imaging technology which has decreased its diagnostic age. High index of clinical suspicion can help in timely diagnosis and management.

## Graphical abstract

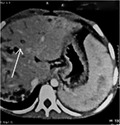

## 1 INTRODUCTION

Congenital saccular dilatation of intrahepatic bile ducts, also known as communicating cavernous ectasia or Caroli's disease, was first described in 1958 by J. Caroli for whom the name of the disease is attributed to.^1^ Type I, characterized by pure cystic dilatations of the intrahepatic bile ducts, is known as Caroli's disease (CD). Type II or complex CD, known as Caroli's syndrome, is characterized by multiple or saccular dilatation of intrahepatic biliary duct (IHBD) in association with congenital hepatic fibrosis.^2^

The incidence of Caroli's syndrome is extremely low and estimated to be one case per 10,00,000 people, with Caroli's disease being even rarer.^3^

Symptoms of Caroli's syndrome include complications of Caroli's disease (bouts of cholangitis, hepatolithiasis, and gallbladder stones) and those of congenital hepatic fibrosis (CHF) and portal hypertension (PH). The main and often the only symptom is unexplained fever without pain or jaundice.^4^ Portal hypertension is not a common and early presentation of the disease.

Here, we present a case of Caroli's syndrome in a five‐year‐old girl with dilatation of IHBD in the right lobe of the liver, bilateral polycystic kidney, and portal hypertension.

## 2 CASE HISTORY

A five‐year‐old female kid presented with complaints of multiple episodes of vomiting of fresh blood for two days and black stool for a day. There was no history of fever, jaundice, abdominal pain, loss of appetite, generalized body swelling, or any previous episodes of similar blood‐mixed vomiting. There was no family history of any liver disease or any cystic kidney disease.

The patient was asymptomatic at the time of the presentation. However, on physical examination, she was pale and had mild abdominal distension with venous dilatations around the umbilicus. The liver was palpable up to 30 cm below the right costal margin. Also, the spleen was palpable 4 cm below the left costal margin and was firm and nontender. Cardiovascular, neurological, and respiratory examinations were unremarkable. Her blood pressure was 120/80 mm Hg which was above the 99th percentile for her age and height according to the WHO chart. (99th percentile for her age and Height of 102.5 cm = 114/78 mm Hg).

On hematological investigation, the patient had hemoglobin of 7.3 gm/dL and RBC of 3.89 millions/μL. Leukopenia and thrombocytopenia were evident with WBC and platelet count of 2500/μL and 88 000/μL, respectively. On peripheral blood smear, anisopoikilocytosis and hypochromia were seen with no evidence of any parasites. The liver function test showed an increase in the alkaline phosphatase level, that is, 376 U/L (normal: <306 U/L) with other results within the normal range. Renal function test results were within normal limits. With the background of pancytopenia, bone marrow aspiration was done which showed normocellular marrow with erythroid hyperplasia without any evidence of atypical cells, hemoparasites, or metastatic deposits.

**Figure 1:**
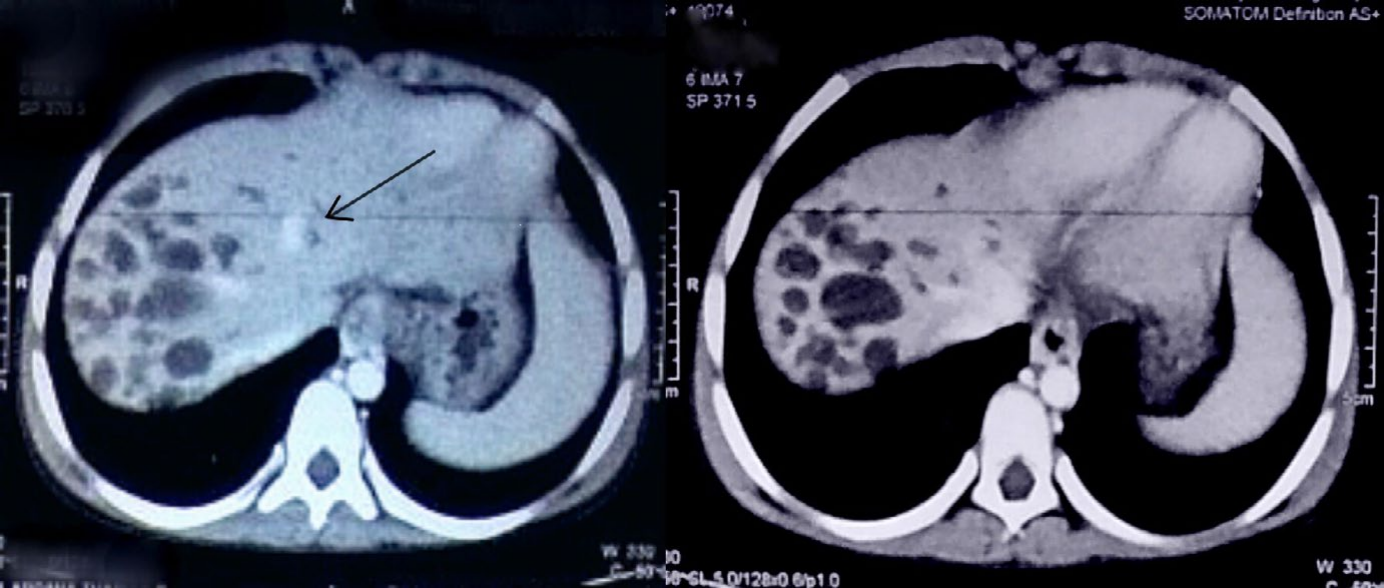
Multiple cystically dilated intrahepatic biliary ducts in segments V, VI, VII, and VIII of the liver. These ducts show no enhancement in post‐contrast images with central enhancing portal radicle giving central dot sign (arrow). However, common bile duct is normal

**Figure 2:**
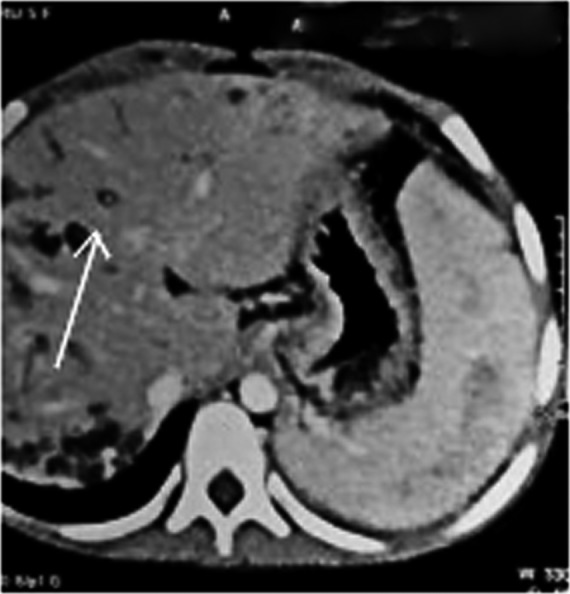
Ducts show no enhancement in post‐contrast images with central enhancing portal radicle giving central dot sign (arrow)

**Figure 3:**
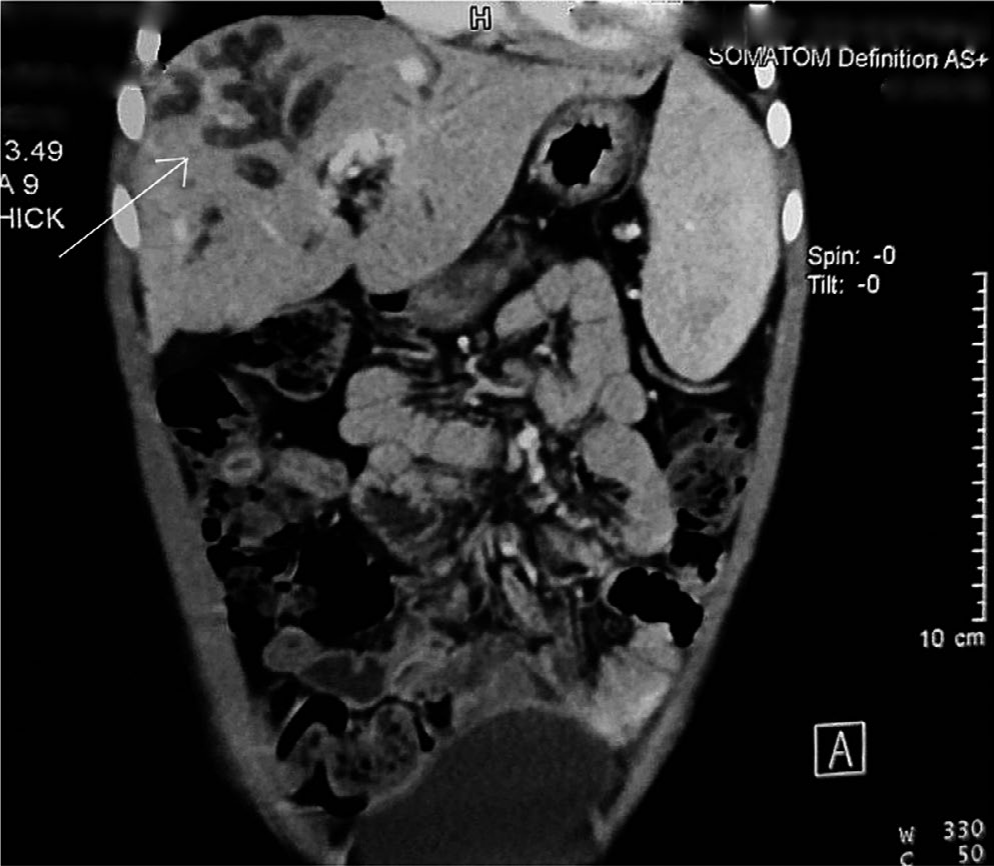
Multiple cystically dilated intrahepatic biliary ducts (arrow)

For evaluation of upper gastrointestinal bleeding with suspected portal hypertension, upper gastrointestinal endoscopy was performed which revealed grade II esophageal varices. Ultrasonography of the abdomen showed multiple fusiform dilatations of intrahepatic biliary ducts within the right lobe of the liver. Bilateral kidney appeared echogenic with loss of corticomedullary differentiation. Computed tomography portogram with urography revealed multiple cystic dilatations of intrahepatic bile ducts predominantly in the right lobe of the liver, the largest measuring approximately 20 × 14 mm in segment VIII, splenomegaly with nonvisualized main portal vein, and multiple collaterals in the hepatic hilum, perisplenic, peripancreatic region with esophageal, and rectal varices along with “central dot” sign in the liver. (Figures 1, 2, and 3).

**Figure 4:**
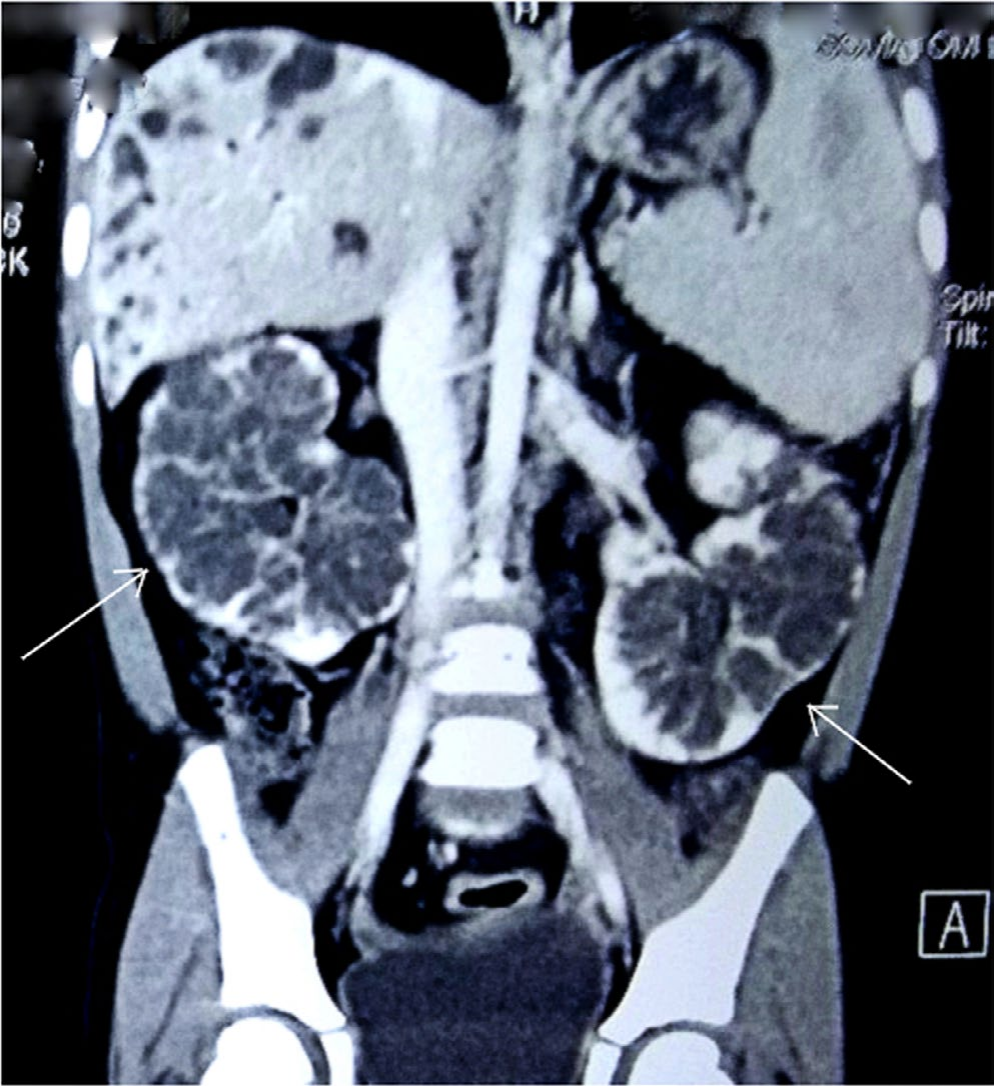
CT scan (coronal view) Multiple variable‐sized cystic lesions are seen (arrows) involving all poles of both kidneys. No calcification, septation, or enhancing solid components seen within the cyst

Similarly, bilateral renal cysts with nephromegaly were seen. (Figure 4) All findings were suggestive of Caroli's disease with bilateral polycystic kidney disease and portal hypertension. After admission, esophageal variceal banding was done and treated prophylactically with propranolol and ranitidine. She was started on amlodipine to control her increased blood pressure. A liver biopsy was not performed. During the hospital stay, the patient received one pint of packed RBC. She was discharged after nine days with no symptoms. She was symptom‐free and doing well on follow‐up after one month and was kept on a regular follow‐up for monitoring the varices, blood pressure, and liver and renal functions.

## 3 DISCUSSION

Caroli's syndrome (CS), originally reported by Jacques Caroli, is a rare congenital disorder characterized by cystic dilatation of the intrahepatic biliary ducts (IHBD) with congenital hepatic portal fibrosis.^5^ There is a distinction between the CS and CD; the latter is characterized by isolated dilatation of large IHBD, the absence of congenital liver fibrosis, and only one portion of the liver affected.^6^

This disease is a developmental condition with probable autosomal recessive inheritance pattern thought to be caused by the complete or partial arrest of ductal plate remodeling (Ductal Plate Malformation) leading to persistence of embryonic biliary ductal structures.^7^ It can affect large as well as small intrahepatic bile ducts that surround the portal tracts which result in cysts surrounding the portal triad.^8^ Various other mechanisms such as neonatal occlusion of hepatic artery and prenatal hepatic vascular accident have also been proposed.^9^ Cytogenetic studies revealed the mutations of PKHD‐1 gene on the chromosome 6p21, which is involved in the synthesis of fibrocystin protein, are responsible for major structural abnormalities of the liver and kidneys.^7^ Studies have indicated that the presence of polycystic kidney disease in a young adult with hepatic fibrosis or Caroli's disease is highly suggestive of autosomal recessive polycystic kidney disease (ARPKD).^10^ Typically, CHF and portal hypertension (PH) do not occur in autosomal dominant polycystic kidney disease (ADPKD).^11^ However, CHF complicated by PH is a rare but potentially life‐threatening complication of ADPKD.^12^ Genetic analysis could not be performed in our case because of financial constraints; thus, the heritance pattern of the cystic kidney could not be identified.

There is high variation in the clinical progression and presentation of Caroli's syndrome, and symptoms may appear early or late during life. Although present from birth, the disease usually remains asymptomatic during the first two decades of life and may also remain so throughout life, necessitating high index of suspicion for its diagnosis.^13^, ^14^ As mentioned, Caroli's syndrome presents a clinical syndrome, a combination of Caroli's disease (bouts of cholangitis, hepatolithiasis, and gallbladder stones), and that of CHF/PH.^15^ A study of 30 patients with congenital IHBD dilatation showed the most frequent initial symptoms of the disease included right upper quadrant pain, fever, anorexia, variceal bleeding (hematemesis, melaena, or hematochezia), fatigue, and jaundice in decreasing order without any difference in frequency of these symptoms between patients with CD and CS.^14^ This shows that variceal bleeding is not an uncommon symptom of the disease condition. However, in CS the clinical presentation is dependent upon the predominant pathology, cholangitis if duct dilatation predominates, and hematemesis/melaena if portal hypertension predominates.^16^ In our patient, portal hypertension predominated with an episode of variceal bleeding and splenomegaly as suggested by history, physical, and endoscopic examination. Associated involvement of kidney (renal tubular ectasia, medullary sponge kidney, cortical cyst, recessive polycystic kidney disease, or rarely autosomal dominant polycystic kidney disease) is usually asymptomatic or may present in infancy (nephrolithiasis, pyelonephritis, or hypertension), while cholangitis and manifestation of portal hypertension are more so in early childhood.^3^ Our patient had no overt signs/symptoms suggestive of kidney involvement except for elevated blood pressure even in the presence of bilateral multiple cysts.

Findings on physical examination include none to hepatosplenomegaly, ascites, jaundice, peripheral edema, hepatomegaly or splenomegaly, features of hepatic insufficiency, and/or portal hypertension.^17^, ^18^ Apart from hepatosplenomegaly and mild abdominal distension, our patient had dilated veins around umbilicus which suggest the presence of portal hypertension for a considerable time period before she was diagnosed. The laboratory findings are nonspecific. Liver function tests are normal except for moderately elevated alkaline phosphatase and gamma‐glutamyl transpeptidase. Our case had elevated alkaline phosphatase level. Also, transaminase levels may be slightly elevated. Findings of splenomegaly or cytopenias secondary to hypersplenism are indicative of portal hypertension as evident in our case. Elevated white blood cell count or erythrocyte sedimentation rate may indicate cholangitis. Associated renal disease can be detected by measuring blood urea nitrogen (BUN) and creatinine values.^4^ Our patient had normal renal function despite the presence of multiple cysts.

Ultrasound or computed tomography is often the first study to demonstrate an abnormally dilated biliary system.^9^ On sonography, the dilated biliary channels are anechoic, and on CT (computed tomography), they are hypodense. The diagnosis rests on the demonstration of continuity of these saccular dilatations with the biliary tree.^19^ Magnetic resonance imaging (MRI) provides better information regarding location, severity, and extent of the disease. The so‐called “central dot sign,” defined as small foci of strong contrast enhancement within dilated intrahepatic ducts, is often found on MRI or CT scan and is considered highly specific for Caroli's syndrome.^20^ The central dot sign along with CT scan and clinical features described helped us make the diagnosis of Caroli's syndrome. Magnetic resonance cholangiopancreatography (MRCP) is considered the most specific and noninvasive examination to depict the multiple ductal dilatations seen in Caroli's disease, called the “lollipop tree” aspect (T2 and most notably T1 sequences after contrast injection).^21^ It shows diverticulum‐like saccule of intrahepatic bile duct ectasia of varying sizes, shapes, and distribution, which communicate freely with the bile duct. Conversely, in Caroli's syndrome, the cystic bile duct ectasia is always smaller (<2 cm) and periportal hepatic fibrosis is seen on T2‐weighted sequences as high signal areas among the portal veins. Both tests could not be performed due to financial constraints of the patient.^18^ However, the histopathological examination of the resected specimen is the definitive investigation and is characterized by hepatic and dense portal fibrosis, along with dilated bile ducts and secondary cirrhosis in some patients with more severe disease.^14^ No invasive procedure was performed in our case.

The differential diagnoses of this entity include von Meyenburg complex, primary sclerosing cholangitis, polycystic liver disease, and choledochal cyst. von Meyenburg complex is a rare condition that usually does not cause symptoms or disturbances in liver function, diagnosed by chance in MRCP, showing multiple small‐size cystic nodules (<1.5 cm) that do not communicate with the biliary tree.^22^

Complications of Caroli's syndrome are cholangitis, sepsis, choledocholithiasis, hepatic abscess, cholangiocarcinoma, and portal hypertension.^23^ After cholangitis occurs, a large number of patients die within 5‐10 years.^19^ The CS patients have more than a 100‐fold increase in the risk of developing cholangiocarcinoma.^24^, ^25^ Death is often related to liver failure or complications of portal hypertension.^23^, ^26^

The treatment of CS is largely supportive and conservative especially in resource‐limited settings like ours. The aim is to treat infections of the biliary tree and complications of portal hypertension, and prevent morbidity. These include antibiotics for infections, stenting, and ursodeoxycholic acid for cholestasis. Measures to treat the portal hypertension include prophylactic beta‐blockers for significant varices and endoscopic banding, sclerotherapy, and shunting for bleeding.^3^ The same (banding and prophylactic therapy) was done in our case. Shunting is generally required for recurrent bleeding episodes not amenable to treatment with sclerotherapy and esophageal tamponade.^27^ In the case of abnormalities located in one lobe, lobectomy may be the best option, which also reduces the risk of developing hepatic malignancy. In diffuse Caroli's disease, treatment options include conservative or endoscopic therapy, internal biliary bypass procedures, and liver transplantation on carefully selected cases. However, liver transplantation represents an uncommon indication for CD or CS.^28^ In addition, if concomitant renal failure ensues from the dysplastic kidneys, liver transplantation combined with renal transplantation might be warranted and is the only curative option. Other complications of polycystic kidney disease like urinary tract infections can be managed with support, and hypertensions tend to respond well to angiotensin‐converting enzyme inhibitors.^8^

## 4 CONCLUSIONS

A definitive diagnosis of Caroli's syndrome is only possible through CT or MRCP or histological examination of the liver specimen, which remains a challenge in the resource‐limited settings. It is important to consider Caroli's syndrome in the differential diagnosis in patients presenting with features of portal hypertension. Delay in treatment can lead to severe life‐threatening hematemesis, cholangitis, or even cancer. Slow and silent progression of the disease warrants regular follow‐up.

## CONFLICT OF INTEREST

None declared.

## AUTHOR CONTRIBUTIONS

SS, SH, and AR: wrote the manuscript. BG and AJ: reviewed the manuscript. All the authors involved in final reviewing of the manuscript.

## INFORMED CONSENT

Written informed consent was taken from the patient's parents before writing the manuscript.
